# FF_483–484_ motif of human Polη mediates its interaction with the POLD2 subunit of Polδ and contributes to DNA damage tolerance

**DOI:** 10.1093/nar/gkv076

**Published:** 2015-02-06

**Authors:** Nadège Baldeck, Régine Janel-Bintz, Jérome Wagner, Agnès Tissier, Robert P. Fuchs, Peter Burkovics, Lajos Haracska, Emmanuelle Despras, Marc Bichara, Bruno Chatton, Agnès M. Cordonnier

**Affiliations:** 1 Biotechnologie et Signalisation Cellulaire, Université de Strasbourg, UMR7242, Illkirch 67412, France; 2 UMR-S1052, Inserm, Centre de Recherche en Cancérologie de Lyon, Lyon 69000, France; 3 Cancer Research Center of Marseille (CRCM), Centre National de la Recherche Scientifique, Unité Mixte de Recherche 7258, Marseille 13009, France; 4 Institute of Genetics, Biological Research Center, Hungarian Academy of Sciences, HU-6726 Szeged, Hungary; 5 Université Paris-Sud, CNRS-UMR8200, Equipe labellisée Ligue Contre le Cancer, Gustave Roussy, Villejuif, France

## Abstract

Switching between replicative and translesion synthesis (TLS) DNA polymerases are crucial events for the completion of genomic DNA synthesis when the replication machinery encounters lesions in the DNA template. In eukaryotes, the translesional DNA polymerase η (Polη) plays a central role for accurate bypass of cyclobutane pyrimidine dimers, the predominant DNA lesions induced by ultraviolet irradiation. Polη deficiency is responsible for a variant form of the *Xeroderma pigmentosum* (XPV) syndrome, characterized by a predisposition to skin cancer. Here, we show that the FF_483–484_ amino acids in the human Polη (designated F1 motif) are necessary for the interaction of this TLS polymerase with POLD2, the B subunit of the replicative DNA polymerase δ, both *in vitro* and *in vivo*. Mutating this motif impairs Polη function in the bypass of both an N-2-acetylaminofluorene adduct and a TT-CPD lesion in cellular extracts. By complementing XPV cells with different forms of Polη, we show that the F1 motif contributes to the progression of DNA synthesis and to the cell survival after UV irradiation. We propose that the integrity of the F1 motif of Polη, necessary for the Polη/POLD2 interaction, is required for the establishment of an efficient TLS complex.

## INTRODUCTION

Lesions that are continuously formed on cellular DNA upon exposure to genotoxic agents impede the progression of the replicative DNA polymerases. One DNA damage response pathway that overcomes replication arrests is translesion synthesis (TLS), in which specialized DNA polymerases allow DNA synthesis along damaged templates, albeit at the cost of reduced fidelity.

The current model for TLS proposes that this multi-step process involves several DNA polymerase switches. In a first step, PCNA, the processivity factor of the replicative polymerases, located at stalled replication forks is monoubiquitinated by the Rad18 ubiquitin ligase (1,2). This post-translational modification promotes the recruitment of Y-family TLS polymerases (Polη, Polι, Polκ and Rev1) by increasing their affinity for PCNA (3–8) and their residence time at sites of DNA damage (9). For most lesions, the bypass is then carried out by a two-polymerase reaction in which insertion of a nucleotide opposite the damage by a particular DNA polymerase is followed by extension by another DNA polymerase (10–12). Polζ, a B-family DNA polymerase, plays this extender role in a process mediated by Rev1, which interacts both with the Rev7 subunit of Polζ and with each of the other Y-family polymerases. Accordingly, recent structural studies provide evidence that Rev1, Polζ and Polκ DNA polymerases cooperate within a megatranslesion polymerase complex (13,14).

Once the lesion bypass is fully completed, the primer terminus is taken over by high-fidelity polymerases of the replicative machinery. It has been shown that the B and C subunits of the replicative DNA polymerase δ (human POLD2 (p50) and POLD3 (p66) or their yeast counterparts Pol31 and Pol32) are also functional components of Polζ (15–17). This observation has led to the proposal that the exchange of the Polδ and Polζ catalytic subunits at the lesion site might occur on a pre-assembled complex of POLD2 and POLD3 proteins bound to PCNA (16). More recently, it has been reported that purified Pol31 and Pol32 can form a complex with the TLS polymerase Rev1 in yeast (18).

Loss of Polη in *Xeroderma pigmentosum* variant (XPV) individuals results in a strong susceptibility to sunlight-induced skin cancers, due to the activation of an error-prone TLS pathway. Polη has a remarkable ability to perform fast and accurate bypass across a *cis*-syn cyclobutane thymine dimer (TT-CPD) *in vitro* (19–21) and *in vivo* (22). Bypass of this particular lesion may thus represent a special case in the TLS model, as Polη is able to perform both the insertion and extension steps without the help of other TLS polymerases (23). Several domains of Polη have been shown to be important for its function in TLS: the PIP and the UBZ domains are involved in the interaction with PCNA and its monoubiquitinated form, respectively (3–5,24). The activity of Polη is also regulated by post-translational modifications such as monoubiquitination (25) or phosphorylation at S601 (26), S587 and T617 (27). In this paper, we investigated whether Polη could directly interact with Polδ. Only POLD2, the B-subunit of Polδ, was found to bind to Polη in a two-hybrid experiment and this interaction was further confirmed in a pull-down assay. Mutation of the FF_483–484_ motif of Polη (designated F1 motif) involved in the interaction with POLD2 reduces the Polη ability to perform TLS catalyzed by cellular extracts *in vitro* and to rescue the UV sensitivity of XPV fibroblasts. Taken together, our results disclose a novel role for the F1 motif of Polη and provide the first evidence for a direct interaction of this TLS polymerase with the replicative polymerase δ. We propose that this interaction facilitates the access of Polη to the DNA substrate.

## MATERIALS AND METHODS

### Cell lines and plasmids

Cells were grown at 37°C in Dulbecco's modified Eagle's medium supplemented with 5% fetal bovine serum (Eurobio) and gentamicin (Sigma). MRC5-V1 cells (called MRC5 in this paper) are SV40-transformed normal human lung fibroblasts (28). The XP30RO cell line (SV40-transformed XPV human fibroblasts) has a homozygous deletion in the Polη gene resulting in a truncated protein of only 42 amino acids (29). XP30RO cell lines expressing wild type (WT) or mutated forms of Polη were generated by transfection with a pcDNA3.1/zeo(-) plasmid harboring the corresponding Polη sequence; transfected cells were thereafter selected with 100 μg/ml of zeocin (Invitrogen). ECFP-Polη and EYFP-Polη constructs have been described elsewhere (30). Mutations in the coding sequence of Polη were generated by site-directed mutagenesis. Full oligonucleotide sequences are available from the authors on request. PIP* corresponds to the FF_708–709_AA mutation in the Polη sequence. DEAD Polη harbors a DE_115–116_AA mutation within the catalytic domain.

### Fluorescence microscopy

MRC5 cells grown on glass coverslips were transfected using the jetPEI reagent according to the manufacturer's protocol (Polyplus). Cells were UV-irradiated at 8 J/m^2^ 24 h after transfection and were processed after 8 h. Cells were washed twice with phosphate buffered saline (PBS) and treated for 5 min with CSK 100 buffer (100-mM NaCl, 300-mM sucrose, 3-mM MgCl_2_, 10-mM Pipes pH6.8, 1-mM EGTA) supplemented with 0.2% Triton-X100 and a protease inhibitor cocktail (Complete, Roche). Cells were washed and then fixed in 4% formaldehyde in PBS and mounted onto slides using Fluorescent mounting medium (SouthernBiotech). Slides were analyzed on a Leica DMRA2 microscope equipped with an Orca-ER CCD camera (Hamamatsu) and the capture software OpenLab 4.1 (Improvision).

The proximity ligation assay (PLA) kit was purchased from Sigma. Cells were washed twice with PBS and treated for 5 min with ice-cold CSK 100 buffer (100-mM NaCl, 300-mM sucrose, 3-mM MgCl_2_, 10-mM Pipes pH6.8, 1-mM EGTA) supplemented with 0.2% Triton-X100 and a protease inhibitor cocktail (Complete, Roche) and fixed as described above. The primary antibodies (1/600 rabbit anti-Polη, H-300 Santa Cruz; 1/500 mouse anti-PCNA, PC10, Santa Cruz; 1/300 goat anti-POLD2, sc-8800, Santa Cruz) were incubated overnight at 4°C. Secondary antibodies conjugated with the PLA-oligonucleotide probes were used (Duolink II PLA probe anti-mouse PLUS, anti-rabbit MINUS and anti-goat PLUS) according to the manufacturer's instructions.

### Clonogenic assay

XPV cell lines expressing WT or mutant Polη from the pcDNA vector were plated in triplicate in 10-cm dishes (3000 cells per plate). The next day, cells were washed in PBS and UV-irradiated at a dose of 8 J/m^2^ (254 nm). Caffeine (0.375 mM) was added to the culture medium and cells were incubated for 10 days. The number of colonies was assessed after fixation in 4% formaldehyde and crystal violet staining.

### Flow cytometry (FACS)

Cells were collected by trypsinization, washed in PGE (glucose 1 g/l in PBS-1-mM ethylenediaminetetraacetic acid (EDTA)) and fixed in 70% ethanol in PBS-Glucose-EDTA (PGE) at 4°C for 1 h. Cells were washed twice in PGE and nuclear DNA was stained with propidium iodide (4 μg/ml; Sigma, St. Louis, MO, USA) in the presence of RNase A (10 μg/ml; Invitrogen) for at least 30 min. Stained cells were analyzed on a FACScalibur (Becton Dickinson, Franklin Lakes, NJ, USA) using CellQuest software. Twenty to thirty thousand cells gated as single cells were analyzed.

### Two-hybrid analysis

Two-hybrid analysis was performed in *Saccharomyces cerevisiae* AH109, as described previously (30). Construction of plasmid pGBKT7 containing POLD2 sequence has been made after amplification using primers 5′-ATGGCCATGGAGATGTTTTCTGAGCAGGCTGCC-3′ and 5′-TCGACGGATCCCTCAGGGGCCCAGCCCCAG-3′. The NcoI/BamHI fragment has been inserted in pGBKT7. Mutations were generated by site-directed mutagenesis into the Polη coding sequence cloned in pACT2 plasmid. Full oligonucleotide sequences are available from the authors on request.

### Proteins

His-GST, His-GST-Polη_393–511_ and His-GST-Polη_393–511_(F1*) were cloned into pETM-30 plasmid, expressed in *Escherichia coli* BL21 (DE3) pLysS bacteria and purified in one step by Nickel column chromatography. Purified proteins were stocked in 50-mM NaPO4 (pH = 7.4), 300-mM Nacl and 10% glycerol containing buffer and flash frozen in liquid nitrogen.

GST-Polη was purified as described previously (31).

For expressing GST-Flag-POLD2, the POLD2 gene was cloned in fusion with glutathione S-transferase (GST) and FLAG tag under the control of the *S. cerevisiae* galactose-inducible phosphoglycerate promoter using the Gateway cloning system (Invitrogen) to generate plasmid BIL2695. GST-Flag-POLD2-expressed yeast cells were disrupted in NTE buffer (20-mM Tris-HCl pH 7.5, 1-mM dithiotreitol, 0.01% Nonidet P-40, 10% glycerol, 1000-mM NaCl, 5-mM EDTA and protease inhibitor mixture). After clarification of the crude extract by centrifugation it was loaded onto a Glutathione-Sepharose column. First, the column was washed with buffer NTE followed by washing with buffer NT (20-mM Tris-HCl pH 7.5, 1-mM dithiotreitol, 0.01% Nonidet P-40, 10% glycerol, 100-mM NaCl). The GST-Flag-POLD2 protein was eluted by Prescission protease cleavage in buffer NT. The Flag-POLD2 containing fractions was pooled and stored at –80°C.

*In vitro* transcription/translation of full-length WT or mutant Polη and POLD2 was performed using a TnT Quick coupled lysate system (Promega) according to the manufacturer's instructions. The expression vectors encoding the full-length Polη (pGBKT7-Polη) or POLD2 (pIVEX POLD2 without His-Tag) were added to the reaction mixture and incubated for 90 min at 30°C.

### Pull-down assays

Purified GST or GST-Polη proteins (1.5 μg) were incubated with Glutathione-Sepharose (GTH) beads for 45 min at 4°C with FLAG-POLD2 (0.5 μg) in binding buffer containing 40-mM Tris HCl, pH 7.5, 70-mM NaCl, 0.1-mM DTT, 0.01% NP40, 10% glycerol). Beads were washed three times with the binding buffer and boiled. Elution fractions were analyzed by 10% sodium dodecyl sulphate/polyacrylamide gel electrophoresis (SDS/PAGE) followed by Coomassie blue staining or immunoblotting using anti-Flag antibody.

Equal amounts (125 μg) of His-GST, His-GST-Polη_393–511_ or His-GST-Polη_393–511_(F1*) were immobilized onto equal amounts (1.32 mg) of pre-washed Dynabeads His-Tag isolation and pull down (Life Technologies). Washed and pre-loaded beads were then mixed with 35 μl of the POLD2 TnT reactions in 0.75x Buffer 1 (Buffer 1: 3.25-mM NaPO_4_ pH = 7.4, 70-mM NaCl, 0.01% Tween20 complemented with complete mini EDTA-free proteases inhibitors, Roche) and incubated for 1.5 h at 4°C with resuspension every 5 min. Beads were washed once with 100 μl of Buffer 1, resuspended in 20 μl of 2x SDS page loading buffer and boiled for 10 min.

### TLS assay

The construction of single-stranded plasmids containing either a single Cyclobutane Pyrimidine Dimer (CPD) lesion (pUCTT-CPD.ss) or a unique G-AAF adduct (pUC3G3-AAF.ss) has been extensively described (32). Primer extension assays were performed as previously described (33). Standard reactions (6.25 μl) were performed using 10 fmol of primed single-strand DNA and an XPV cell extract (20 μg) supplemented with WT or mutated Polη (0.2 μl of reticulocyte lysates). The quantification of the levels of TLS was determined using the ImageQuant TL software after phosphorimaging (GE Healthcare). The percentage of TLS was calculated as the ratio of the intensity of the bands of non-slipped TLS (TLS 0) or slipped TLS (TLS-1) to the sum of the intensity of the TLS and L-1 bands.

### Immunoblotting

Proteins were loaded onto 8 or 10% SDS/polyacrylamide gels. After electrophoresis, separated proteins were transferred onto a PVDF membrane (Biorad) and probed with antibodies against Polη (H300, sc-5592), Polδ (A9, sc-1777), POLD2 (C-20, sc-8800), c-Myc (9E10, sc-40) from Santa Cruz Biotechnology or GST (G7781) from Sigma.

## RESULTS AND DISCUSSION

### DNA Polη interacts with POLD2, the Polδ subunit B

Human Polδ is a replicative DNA polymerase consisting of four subunits (POLD1, POLD2, POLD3 and POLD4). POLD2 serves as a scaffold for the assembly of Polδ by interacting simultaneously with all of the other three subunits. Using a yeast two-hybrid assay (Y2H) we tested the ability of Polη fused to the GAL4-activation domain to interact with each of the four Polδ subunits fused to the GAL4-DNA binding domain and found that only POLD2 interacts with Polη. No growth was observed in strains expressing both the Polη and empty expression plasmids, confirming the specificity of the Polη/POLD2 interaction.

**Figure 1.**
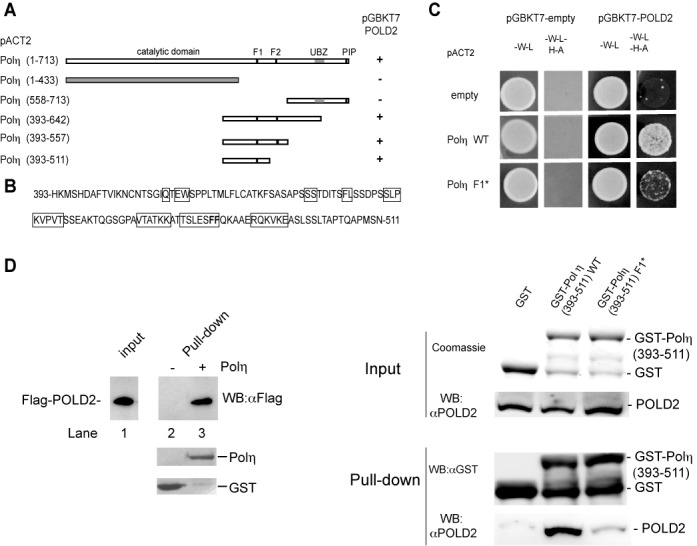
Polη interacts with the POLD2 subunit of Polδ. (**A**) Polη full length or truncation mutants and POLD2 proteins were expressed in the yeast strain AH109 as a transcription activation domain fusion protein (in pACT2) and a DNA binding domain fusion protein (in pGBKT7), respectively. Yeast transformants expressing both Polη and POLD2 fusion proteins are selected on double drop out medium (-W-L). Positive interactions are indicated by growth on quadruple drop out medium (-W-L-A-H). (**B**) Minimum amino acid sequence of the Polη region that interacts with POLD2. Residues that were mutated to alanine in the full-length Polη coding sequence and tested for their interaction with POLD2 are boxed. (**C**) Wild type and FF_483–484_AA (Polη F1*) of full-length Polη were examined for the interaction with POLD2. (**D**) Association of POLD2 with Polη *in vitro*. Left panel: physical interaction between the purified human DNA polymerase η and the POLD2 subunit of DNA polymerase δ. GST pull-down experiment was carried out using Flag-POLD2 and GST-Polη or GST followed by immobilization on GTH beads. The bound proteins were analyzed by immunoblotting or Coomassie blue staining. Right panel: selective binding of POLD2 with His-GST-Polη_393–511_. Pull-down experiments were carried out using *in vitro* translated POLD2, His-GST, His-GST-Polη_393–511_ or His-GST-Polη_393–511_ (F1*) followed by immobilization on IMAC magnetic beads. Input and bound proteins were analyzed by Coomassie blue staining or immunoblotting. Inputs correspond to 1/10th the protein amount used for pull-down.

Further analysis using the same Y2H assay revealed that the region of Polη spanning amino acids 393–511 was sufficient for the binding to POLD2 (Figure 1A). In order to identify key residues of Polη involved in the interaction with POLD2, we performed an alanine scanning analysis of several amino acid stretches (boxed in Figure 1B) within this region (393–511). Among the selected sequences, only mutation of the TSLESFF sequence in Polη strongly diminishes the interaction with POLD2. Further mutational analysis within the TSLESFF stretch revealed that the FF motif (FF_483–484_, designated F1) within this region is essential for the binding to POLD2 (Supplementary Figure S1A and Figure 1C). The direct interaction of Polη with POLD2 and the contribution of the F1 motif to this interaction was confirmed using pull-down assays (Figure 1D). Both full-length Polη_1–713_ and Polη_393–511_ fragment interact *in vitro* with POLD2, while the mutated F1 version of Polη_393–511_ looses this ability.

Together, these results show that Polη binds to POLD2 via the fragment spanning amino acids 393–511 and that the integrity of the F1 motif within this sequence is essential for the Polη/POLD2 interaction. No interaction of POLD2 with Polκ and Polι was detected by the Y2H assay (Supplementary Figure S1B), suggesting that POLD2 specifically interacts with Polη, which thus may play a unique role within the replication complex in higher eukaryotes.

### The F1 motif of Polη contributes to lesion bypass *in vitro*

Using single-stranded monomodified plasmids, we have previously shown (33,34) that in cell-free extracts, TLS opposite an *N*-2-acetylaminofluorene guanine adduct (G-AAF) or a *cis*-syn cyclobutane thymine dimer (TT-CPD) is dependent on a catalytically active Polη. Even though the primer extension assay employs an ssDNA template, we demonstrated that it recapitulates the successive DNA polymerase switches that are necessary to promote TLS. In this assay, the radiolabeled primer located 91 nucleotides away from the lesion site is extended in a Polδ-dependent manner up to the lesion site (L-1; data not shown). We have clearly established that PCNA as well as the PIP and UBZ domains of Polη that mediate the interaction with PCNA and Ubiquitin, respectively, both contribute to the TLS reaction (34).

**Figure 2.**
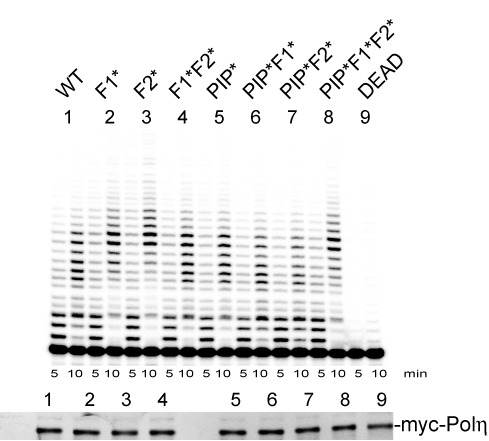
Wild-type or mutant Polη displays the same catalytic activities. Time course of DNA synthesis catalyzed by wild-type (WT) or mutant Polη using a primed single-stranded template (pUC118.ss). Upper panel: DNA products were subjected to electrophoresis on a 20% polyacrylamide–7-M urea denaturing gel. Lower panel: immunoblot of the different forms of Polη produced in rabbit reticulocytes lysates, using an anti-myc antibody.

**Figure 3.**
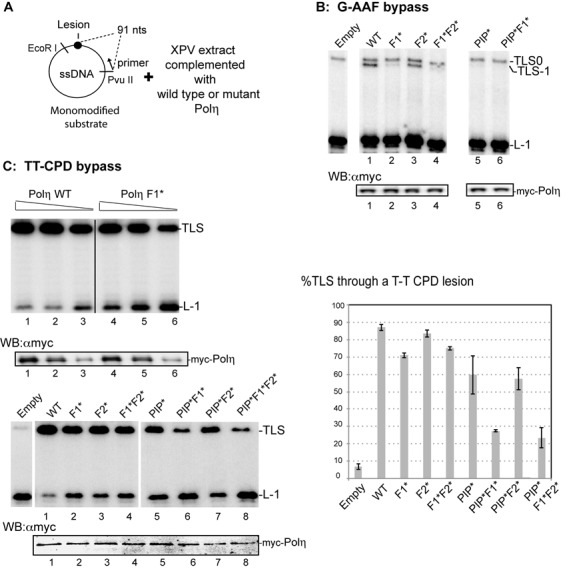
The F1 motif of Polη contributes to TLS *in vitro* through an AAF adduct and a TT-CPD lesion. (**A**) Outline of the experiment and diagram of the modified plasmids. The length of the strand produced upon elongation of the [32P]-labeled primer, up to the lesion site, is indicated. nts : nucleotides. (**B**) TLS efficiency through a G-AAF adduct located on the third guanine of a run of 3G. Monomodified DNA substrates (10 fmol) were incubated 20 min at 37°C in the presence of XPV cell-free extracts (20 μg) complemented with an equal amount (0.2 μl) of different forms of Polη produced *in vitro*. Samples were analyzed by electrophoresis through an 8% denaturing polyacrylamide gel. Product L-1 is generated when synthesis is blocked one nucleotide before the lesion. TLS0 and TLS-1 are TLS products through an AAF adduct. Below the gel: immunoblot of the different forms of Polη produced in rabbit reticulocytes lysates added to the reactions. (**C**) TLS efficiency through a TT-CPD lesion. Upper panel: monomodified DNA substrates (10 fmol) were incubated 10 min at 37°C in the presence of various amounts (0.33, 0.25 and 0.125 μl) of either Polη wild-type (WT) or Polη mutant (Polη F1*) mixed with XPV cell-free extracts (20 μg). Below the gel: immunoblot of the different forms of Polη produced in rabbit reticulocytes lysates added to the reactions. Lower panel: monomodified DNA substrates (10 fmol) were incubated 10 min at 37°C in the presence of XPV cell-free extracts (20 μg) complemented with the same amount (0.2 μl) of different forms of Polη produced *in vitro*. Below the gel: immunoblot of the different forms of Polη produced in rabbit reticulocytes lysates and present in the reactions. Right panel: quantitative analysis of TLS efficiency with the different versions of Polη. Error bars denote the standard deviation (SD) of at least two experiments performed with independent Polη samples (0.2 μl).

Besides the PIP and UBZ domains, the F1 motif has previously been shown to be involved in the interaction between Polη and Rev1 in Y2H (35) together with a second FF motif located downstream in the sequence (FF_531–532_, designated F2). However, recent reports (14,36) demonstrated that the specific intermolecular interactions between Polη and Rev1 more specifically involved the F2 motif as part of the Rev1-interacting motif conserved between Polη, Polι and Polκ (RIM: FFxxK). To investigate the roles of these FF motifs in the TLS activity of Polη, we expressed the WT and mutant F1, F2 and PIP proteins (designated F1*, F2* and PIP*), or combinations of them, in rabbit reticulocyte lysates. In order to verify that the catalytic activities of these proteins were equivalent, we tested their primer extension efficiencies on undamaged DNA templates (Figure 2). We then tested the ability of these proteins to complement an XPV cell extract for the bypass of a G-AAF adduct and a TT-CPD lesion (Figure 3A). Note that a single XPV extract is used for different experiments, making the TLS efficiency among the various forms of Polη readily comparable.

When the AAF adduct is located at the third guanine of the 5′-GGG-3′ target sequence, the bypass catalyzed by Polη predominantly induces a -1 frameshift mutation (TLS-1) (33,37). Figure 3B shows that the Polη F1* and F1*F2* mutant polymerases are clearly unable to complement the XPV cell extracts to generate the Polη-specific TLS-1 product, whereas a mutation of the F2 motif has no effect. Consistently with previous data, the bypass across the G-AAF adduct is strictly dependent on the PIP motif of Polη (34). Similarly, the TT-CPD bypass efficiency of the complemented extract has been tested (Figure 3C). As compared to the reaction catalyzed by the WT enzyme, complementation of the XPV extract with Polη F1* results, in a reproducible manner, in an increased accumulation of L-1 product, clearly indicating a defect of this mutant in the TT-CPD bypass. This defect is further highlighted when the PIP* and F1* mutations are combined. On the other hand, and as observed for the G-AAF adduct, the F2* mutation (alone or in combination with PIP*) does not affect the TT-CPD bypass activity of Polη in this assay. Altogether, these results show that the F2 motif of Polη, known to be involved in the interaction of Polη with Rev1, is not necessary for TLS in cellular extracts, suggesting that Rev1 does not play any role in TLS through a TT-CPD lesion and a G-AAF adduct when assayed *in vitro*. We confirmed this hypothesis by showing that both WT and Rev1^−/−^ cell extracts (38) display similar TLS efficiencies through G-AAF or TT-CPD (Supplementary Figure S2). In agreement with this result, CPD bypass *in vivo* has also been shown to be Rev1 independent in mouse cells (39).

We have previously shown that PCNA is monoubiquitinated during the primer extension reaction catalyzed by cell-free extracts (40). We hypothesize that the presence of monoubiquitinated PCNA (Ub-PCNA) at the template/primer junction, thought to increase the residence time of Polη at the lesion site, could compensate for the effect of the F1* mutation on TLS efficiency. To test this hypothesis, extracts from XPV cells were depleted of PCNA and then complemented with either a WT or a non-ubiquitinable form of purified PCNA (K164R mutated PCNA). As expected, endogenous PCNA and purified WT PCNA, but not K164R PCNA, were monoubiquitinated during the primer extension reaction (Supplementary Figure S3A). We observed that the efficiency of bypass is globally lower in PCNA complemented cell-free extracts as compared to the non-treated extracts (compare Figure 3C and Supplementary Figure S3B), presumably because the depletion procedure alters some protein function. Nevertheless, the bypass deficiency of WT or Polη F1* is barely affected by the ubiquitination status of PCNA, arguing that the role of the F1 motif differs from that of Ub-PCNA for the bypass of a TT-CPD lesion assayed *in vitro* (Supplementary Figure S3B).

### The F1 motif of Polη is essential for cell survival and cell cycle progression but not for foci formation after UV irradiation

**Figure 4.**
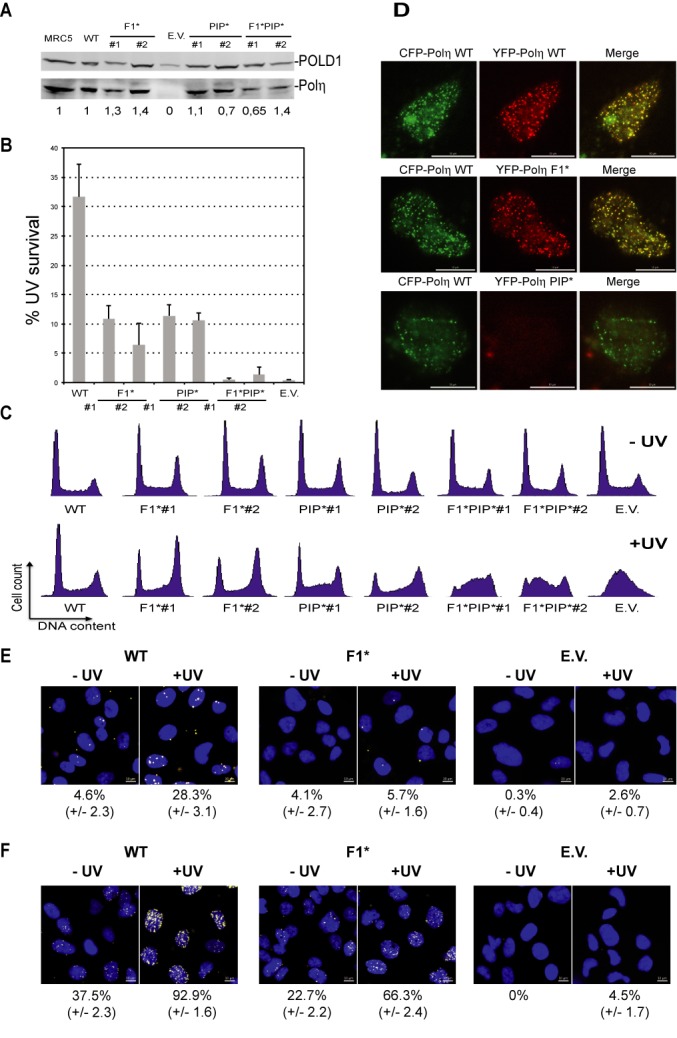
The F1 and the PIP motifs of Polη both contribute to cell survival and cell cycle progression following UV irradiation. (**A**) Immunoblots showing the expression of ectopic Polη versions in the different XPV-complemented cell lines. POLD1 was used as a loading control. The values under the immunoblot represent the ratio of the intensity of Polη to POLD1 bands, relative to MRC5. (**B**) Clonogenic survival assay with XPV-derived cell lines expressing Polη wild type or mutant following UV irradiation (8 J/m^2^) and growth in 0.375-mM caffeine-containing medium (EV: empty vector). Error bars represent SD from at least three independent experiments. (**C**) Cell cycle profiles of XPV cells complemented with the indicated wild-type and mutant forms of Polη determined by flow cytometry after DNA staining with propidium iodide. (**D**) Representative images of CSK-extracted nuclei from MRC5 cells cotransfected with the indicated plasmids and UV-irradiated (8 J/m^2^). Scale bar = 10 μm. Representative images of one of the three independent PLA assays for Polη/POLD2 (**E**) or Polη/PCNA (**F**) interactions. XPV cells complemented with the indicated wild-type and mutant forms of Polη were either non-treated or UV-irradiated (20 J/m^2^) and processed after 6 h. CSK-extracted nuclei were labeled with DAPI (blue). PLA signals were observed as white dots. The percentage of positive cells (containing at least four fluorescent spots) was scored (more than 450 cells were examined for each condition in panel (E) and more than 300 cells in panel (F)). The mean values of two (panel (F)) or three independent experiments (panel (E)) ±SD are indicated.

Having shown that the F1 motif of Polη is required for its interaction with POLD2 as well as for TLS *in vitro*, we sought to determine the role of this motif in UV-induced DNA damage tolerance *in cellulo*. XPV cell lines expressing either WT or mutated Polη were stably established, and clones that express levels of Polη similar to the endogenous level of Polη in normal immortalized MRC5 fibroblasts were selected (Figure 4A). The UV survival of two independent cell lines expressing the Polη F1* is lower than that of cell lines expressing WT Polη (Figure 4B). A previous report (41) showed that a mouse Polη mutated in the F1 motif (FF_482–483_AA) fully corrects the UV sensitivity of the parental XP-V cells to the level of WT cells. In this study, the complemented cells express high levels of Polη (more than 5-fold over the endogenous level). Such high-expression levels make fine regulation mechanisms dispensable as already shown for mutations in PIP or UBZ domains of Polη (34,42). As observed *in vitro*, the effect of the F1 mutation on cell survival is highly strengthened when F1* is coupled to PIP*. Indeed, cells expressing the Polη F1*PIP* double mutant are shown to be much more sensitive to UV irradiation than those expressing either the Polη F1* or PIP* single mutants. The same marked decrease in UV survival of double mutants compared to single mutants has already been described for cells harboring Polη UBZ*PIP* and Polη S601APIP* (25,26,34,42).

As shown in Figure 4C, all cell lines tested displayed a similar cell cycle distribution before UV irradiation. However, cells expressing WT or single mutant Polη showed a significant blockage in S/G2 phases 24 h after UV irradiation, in agreement with other studies (26). Remarkably, cells expressing Polη F1*PIP* exhibit a phenotype similar to XPV cells as they appear to be completely blocked in S phase.

We compared the efficiency of replication foci formation of WT and mutant Polη. For that purpose, we transiently co-expressed WT ECFP-Polη and either WT or mutated EYFP-Polη in human fibroblasts (MRC5). We observed a nearly perfect colocalization of ECFP and EYFP signals (Figure 4D) in cells expressing WT ECFP-Polη and EYFP- Polη F1* 8 h after UVC exposure (8 J/m^2^). In contrast, a mutation of the PIP motif abrogates the relocalization of Polη in foci (Figure 4D), as already reported (42). This result indicates that the mutation of the F1 motif of Polη does not alter the function of PIP domain, clearly indicating that the two domains are required for separate functions of Polη.

Finally, we used the PLA to investigate the Polη/POLD2 interaction in XPV cells stably expressing WT or mutant Polη at physiological levels. While the interaction signal was barely detectable in the chromatin of non-irradiated cells expressing WT Polη, a strong positive signal is observed 6 h after UV irradiation (20 J/m^2^). This interaction signal between Polη and POLD2 is abolished in UV-irradiated cells stably expressing Polη F1*, showing that, when Polη is not overexpressed, F1 motif is required to maintain Polη and POLD2 in close proximity within the replication complex. To further analyze the role of the F1 motif in the stabilization of Polη within replication complexes, we used the PLA assay to analyze Polη/PCNA interaction (Figure 4F). For both cells expressing either Polη WT or Polη F1*, the fraction of positive cells increases similarly (2.5–2.9-fold) upon UV irradiation, indicating that the F1 motif mutation does not affect the recruitment of Polη. However, the interaction signal between Polη and PCNA is systematically reduced in cells expressing Polη F1*, as compared to cells expressing Polη WT, confirming that F1 motif contributes to the stability of Polη within the replication complexes.

Clearly, the assay detects more Polη/PCNA than Polη/POLD2 interactions in cells expressing Polη WT or Polη F1*. Different hypotheses, or combinations of them, may explain this difference. First, the quality of the antibodies used may largely impact the signal-to-noise ratio. Second, the POLD2/Polη interaction may be more transient than the PCNA/Polη one. Finally, it is conceivable that the POLD2/Polη interaction only occurs in a fraction of replication complexes.

## CONCLUSION

Using both *in vitro* and *in vivo* approaches, we show in this work that the F1 motif of Polη contributes to generate a fully functional Polη. Furthermore, the double F1* PIP* mutation has a much more dramatic effect than either single mutation on the efficiency of TLS *in vitro* across a T-T CPD lesion and on the survival of UV-irradiated cells. In agreement with the survival data, the cells expressing Polη F1*PIP* were severely blocked in S phase 24 h after UV irradiation, similarly to XPV cells.

These defects indicate that F1 and PIP motifs differentially contribute to the full activity of Polη. It has previously been observed that the UBZ domain contributes to the retention of Polη at sites of stalled replication forks. As a consequence, a Polη PIP*UBZ* double mutant failed to rescue the UV sensitivity of XPV cells (34,42). Here we provide evidence that the F1 motif is additionally required for efficient TLS *in vitro*, even in the presence of Ub-PCNA. Moreover, while the F1 motif is not necessary for Polη foci formation it nevertheless contributes to a great extent to UV survival. This result indicates that the F1 motif plays an essential role in TLS, which is not interchangeable with that of Polη/PCNA or Polη/Ub interactions. Polη/POLD2 interaction via the F1 motif may provide a complementary anchor point facilitating either Polη integration into the replisome complex or its access to the template/primer junction in a productive conformation.

Human Polδ, the major lagging strand DNA polymerase, is a heterotetramer composed of POLD1, POLD2, POLD3 and POLD4 subunits. This DNA polymerase is also likely responsible for filling the lesion-induced post replicative gaps, although this remains to be established. It is interesting to note that the POLD4 subunit, which binds to both POLD1 and POLD2, is degraded in response to DNA damage (for a review see (43)). This observation led to the proposal of a working model for the Polδ/Polη switching mechanism on PCNA (44). In this model, when Polδ encounters a DNA damage, loss of POLD4 results in the disengagement of POLD1 from the primer terminus, which becomes accessible for Polη. Determining whether the F1 motif is required for TLS at stalled replication forks to fill post-replicative gaps (in a situation where POLD4 is absent from Polδ) or at processive ongoing replication forks (when POLD4 is present) may help to decipher the molecular basis of TLS regulation. For what concerns the lesions encountered during processive leading strand DNA synthesis, the mechanisms of Polη access to the DNA substrate remains to be investigated.

In conclusion, our work provides evidences for a direct interaction between Polη and POLD2 mediated by the F1 motif of the TLS polymerase and for a major role of this motif in both TLS *in vitro* and UV survival of human cells. Given the recent findings that POLD2 and POLD3 subunits of Polδ are functional components of Polζ (15–17), we consider that the Polη/POLD2 interaction described here may also be involved in the switch between these two TLS polymerases when they are involved together in the bypass of specific lesions (45,46).

## SUPPLEMENTARY DATA

Supplementary Data are available at NAR Online.

SUPPLEMENTARY DATA
