# Supplementary material for: FF483–484 motif of human Polη mediates its interaction with the POLD2 subunit of Polδ and contributes to DNA damage tolerance
Source: Nucleic Acids Res. 2015 Feb 6;43(4):2116–25. doi: 10.1093/nar/gkv076 (PMC4344513; doi:10.1093/nar/gkv076)
Supplement: SUPPLEMENTARY DATA [file supp_43_4_2116__index.html]

FF483–484 motif of human Polη mediates its interaction with the POLD2 subunit of Polδ and contributes to DNA damage tolerance — FF483–484 motif of human Polη mediates its interaction with the POLD2 subunit of Polδ and contributes to DNA damage tolerance — SUPPLEMENTARY DATA 

# FF483–484 motif of human Polη mediates its interaction with the POLD2 subunit of Polδ and contributes to DNA damage tolerance

## SUPPLEMENTARY DATA

**Files in this Data Supplement:**

- SUPPLEMENTARY DATA
