# Supplementary material for: FF483–484 motif of human Polη mediates its interaction with the POLD2 subunit of Polδ and contributes to DNA damage tolerance
Source: Nucleic Acids Res. 2015 Feb 6;43(4):2116–25. doi: 10.1093/nar/gkv076 (PMC4344513; doi:10.1093/nar/gkv076)
Supplement: SUPPLEMENTARY DATA [file supp_gkv076_nar-02676-d-2014-File006.pdf]

Supplementary Materials

Supplementary Fig. 1

A: Alanine scanning analysis of Polη

B: Polκ and Polι do not interact with POLD2

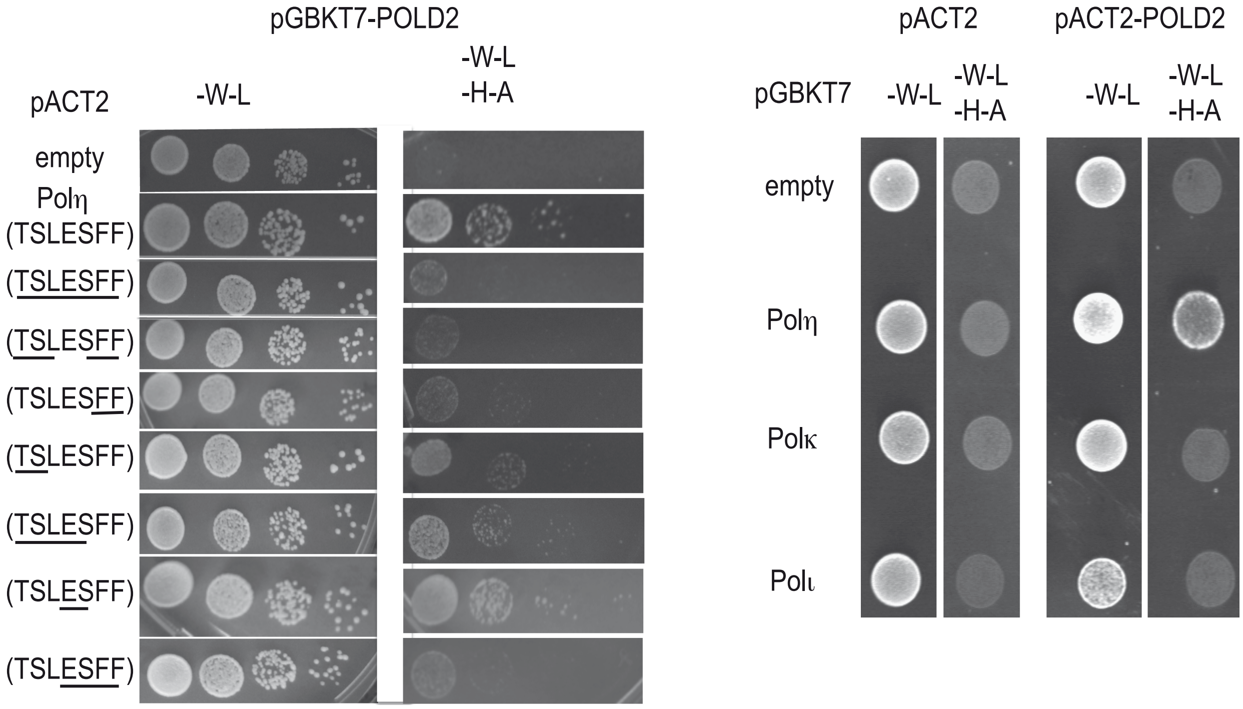

Supplementary Fig. 1:

Polη, Polκ, Polι and POLD2 proteins were expressed in the yeast strain AH109 in Y2H vectors as indicated. Yeast transformants expressing both activation and DNA binding domains fusion proteins are selected on double drop out medium (-W-L). Positive interactions are indicated by growth on quadruple drop out medium (-W-L-A-H). A**:** Underlined residues were mutated to alanine in the full-length Polη coding sequence. Serial dilution of the yeast culture is shown (1/10; 1/100; 1/1000).

Supplementary Fig. 2

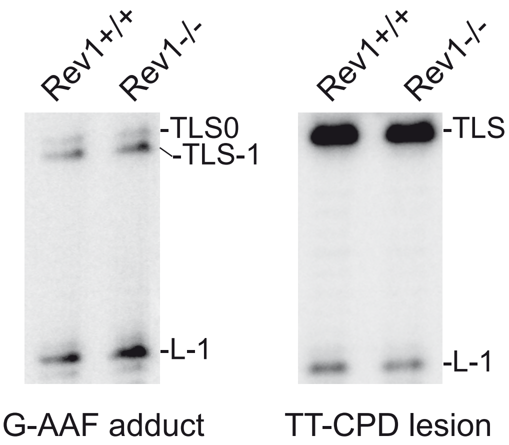

Supplementary Fig. 2: Translesion synthesis catalyzed by cellular extracts *in vitro* is independent on the Rev1 protein

TLS oppposite a G-AAF adduct and a TT-CPD lesion catalyzed by extracts from Rev1+/+ or Rev1-/- MEF cells. DNA products catalyzed by cell extracts (20 μg) were subjected to electrophoresis on an 8% polyacrylamide–8 M urea denaturing gel. L−1 are products generated when synthesis is blocked one nucleotide before the lesion. TLS0 and TLS−1 are products from TLS via nonslipped and slipped intermediates, respectively.

Supplementary Fig. 3

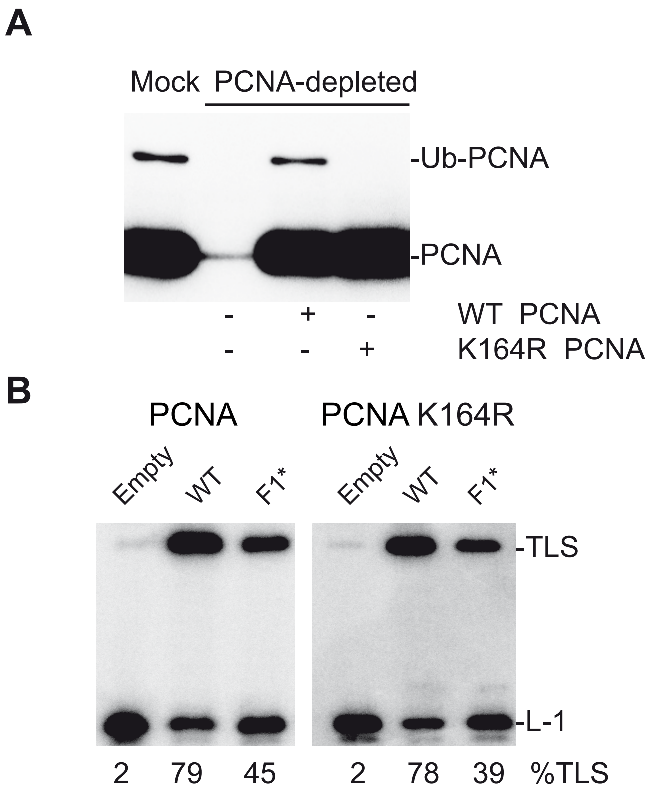

Supplementary Fig. 3: Analysis of Ub-PCNA dependence of the TT-CPD bypass XPV cell-free extracts complemented with either wild type or mutant Polη

A: Mock depleted (M) or PCNA-depleted XP30RO cell extracts (20 μg) were incubated 10 min at 37°C in the presence of 10 fmoles of unmodified substrates in a final volume of 6.25 μl, as indicated. Recombinant PCNA wild type (WT) or PCNA K164R (60 ng) was added to the reactions, as indicated. Aliquots of the samples were analysed by immunoblotting with an anti-PCNA antibody.

B: TLS efficiency through a TT-CPD lesion: Monomodified DNA substrates (10 fmoles) were incubated 10 minutes at 37°C in the presence of either Polη wild type (WT) or Polη mutant (Polη F1*) mixed with PCNA depleted XPV cell-free extracts (20 μg) complemented with PCNA or PCNA K164R as indicated. Products were analyzed by electrophoresis through a 8% denaturing polyacrylamide. L−1 are products generated if synthesis is blocked one nucleotide before the lesion.
